# A Putative Role of Apolipoprotein L1 Polymorphism in Renal Parenchymal Scarring Following Febrile Urinary Tract Infection in Nigerian Under-Five Children: Proposal for a Case-Control Association Study

**DOI:** 10.2196/resprot.9514

**Published:** 2018-06-14

**Authors:** Emmanuel Ademola Anigilaje

**Affiliations:** ^1^ Nephrology Unit, Department of Paediatrics College of Health Sciences University of Abuja Abuja Nigeria

**Keywords:** child, humans, cicatrix, apolipoprotein L1, research design, urinary tract infections, kidney

## Abstract

**Background:**

Although urinary tract infection (UTI) resolves with prompt treatment in a majority of children, some children, especially those aged less than 5 years, also develop renal parenchymal scarring (RPS). RPS causes high blood pressure that may lead to severe chronic kidney disease and end-stage renal disease (ESRD). Although the risk of UTI is higher in white children than in black children, it is unknown whether RPS is more common in white children than in black children as data are scarce in this regard. A common genetic predisposition to kidney disease in African Americans and the sub-Saharan African blacks is the possession of apolipoprotein L1 (APOL1). APOL1 risk variants regulate the production of APOL1. APOL1 circulates in the blood, and it is also found in the kidney tissue. While circulating, APOL1 kills the trypanosome parasites; an increased APOL1 in kidney tissues, under the right environmental conditions, can also result in the death of kidney tissue (vascular endothelium, the podocytes, proximal tubules, and arterial cells), which, ultimately, is replaced by fibrous tissue. APOL1 may influence the development of RPS, as evidence affirms that its expression is increased in kidney tissue following UTI caused by bacteria. Thus, UTI may be a putative environmental risk factor responsible for APOL1-induced kidney injury.

**Objective:**

The aim of this proposal was to outline a study that seeks to determine if the possession of two copies of either G1 or G2 APOL1 variant increases the risk of having RPS, 6 months following a febrile UTI among Nigerian under-five children.

**Methods:**

This case-control association study seeks to determine whether the risk of RPS from febrile UTI is conditional on having 2 APOL1 risk alleles (either G1 or G2). Cases will be children with a confirmed RPS following a febrile UTI. Controls will be age-, gender-, and ethnic-matched children with a febrile UTI but without RPS. Children with vesicoureteral reflux and other congenital anomalies of the urinary tract are to be excluded. Association between predictor variables (ethnicity, APOL1 G1 or G2, and others) and RPS will be tested at bivariate logistic regression analyses. Predictors that attained significance at a *P* value of ˂.05 will be considered for multiple logistic regressions. Likelihood-based tests will be used for hypothesis testing. Estimation will be done for the effect size for each of the APOL1 haplotypes using a generalized linear model.

**Results:**

The study is expected to last for 3 years.

**Conclusions:**

The study is contingent on having a platform for undergoing a research-based PhD program in any willing university in Europe or elsewhere. The findings of this study will be used to improve the care of African children who may develop RPS following febrile UTI.

**Registered Report Identifier:**

RR1-10.2196/9514

## Introduction

### Background

Approximately 7% to 8% of girls and 2% of boys have a urinary tract infection (UTI) during the first 8 years of life [1]. Although febrile UTIs have the highest incidence during the first year of life in both sexes, nonfebrile UTIs occur predominantly in girls older than 3 years [1]. Renal parenchymal scarring (RPS) is a common complication of febrile UTI, and it occurs in 10% to 30% of children with febrile UTIs in developed countries [2]. RPS is a precursor of poor renal growth, hypertension, chronic kidney disease (CKD) and end-stage renal disease (ESRD) that are seen in adulthood [2]. Although RPS is less frequently encountered in developed countries because of improvement in overall health care and close follow-up of children with UTI [2], most cases of UTI in developing countries, including Nigeria, are still being missed, and follow-up of children for RPS is virtually nonexistent [3]. The prevalence of RPS complicating febrile UTI in Nigerian children, therefore, remains unknown. Although the incidence of ESRD is unknown in Nigeria, there is a strong indication that hypertension contributes substantially to it [4-6]. The overall prevalence of hypertension among Nigerians ranges from 0.1% to 17.5% in children and 2.1% to 47.2% in adults [7]. This study becomes relevant as UTI or RPS may have been a precursor of hypertension in the Nigerian population.

The clinical features of UTI in childhood are often different from those found in adults and are frequently nonspecific [3]. Therefore, many cases of UTI are missed, especially in under-fives, among whom the risk of RPS is even greater [2,3,8]. Some children with UTI present only with fever without a clear source of infection or systemic symptoms [9]; fever is also the commonest symptom of UTI [10]. In addition, the presence of another source of fever does not exclude the probability of UTI [8]. Although the available Nigerian studies have reported the prevalence of UTI among febrile under-five children to be between 9% and 37.1%, longitudinal follow-up to determine the risk of renal scars and other complications of febrile UTI is unknown [3,11,12]. This may also be because of lack of imaging modalities such as the dimercaptosuccinic acid (DMSA) scan. DMSA scan is the gold standard for diagnosing RPS [9].

Although the pathogenic mechanism of renal scarring following acute pyelonephritis (APN) is not well understood, the combined presence of congenital anomalies (vesicoureteral reflux, VUR and renal hypodysplasia), inflammation seen in pyelonephritis, and genetics may interact to result in scar formation [13]. The host innate immune response serves as the primary factor for the defense of the kidney [14]. The innate immune system of the kidney in response to invading bacteria involves peptides with antimicrobial properties, renal epithelial cells, cytokines and chemokines, neutrophils, and pattern recognition receptors (PRRs) [14]. The most studied PRR is the toll-like receptor 4 (TLR4), which upon recognizing gram-negative bacteria’s lipopolysaccharide, signals intracellular pathways that leads to transcription of proinflammatory molecules, including chemokines and cytokines, which then activate and target neutrophils to the site of infection [13-18].

These chemokines and cytokines include the interleukin 6, chemokine ligand 2 (CXCL2), interferon β1, CXCL8 (also known as interleukin 8), the interferon regulatory factor 3 (IRF3), interferon-gamma (IFN-γ), tumor necrosis factor (TNF), transforming growth factor beta (TGF-β), and vascular endothelial growth factor (VEGF) [13-18]. TGF-β and VEGF are particularly notable for RPS as they modulate the glomerular and tubule-interstitial scarring. While TGF-β mediates progression of renal fibrosis in association with activation of angiotensin II (ANG II), VEGF enhances the proliferation of vascular endothelial cells, angiogenesis, and microvascular permeability [19-21]. Thus, although bacteria play a crucial role in initiating an inflammatory response during renal colonization, renal damage from APN seems to be primarily because of an inflammatory response of the host rather than an invasion of pathogens [13].

With the exception of antimicrobial peptides and epithelial cells that have not been well studied, single gene defects or variations in various genes have been shown to affect most of the other innate immune expression and response to invading bacteria [14]. For example, a reduced CXCR1 expression is a risk factor for APN, and variations in the CXCR2 receptor and the CXCL8 chemokine lead to UTI susceptibility [22,23]. Low TLR4 expression and signaling protects the host against symptomatic UTI and promotes the development of asymptomatic bacteriuria [24,25]. IRF3 _925 A/G and _776 C/T polymorphisms involved in neutrophil recruitment are strongly associated with febrile UTI susceptibility [26]. Genetic polymorphisms in both TGF-β and VEGF are also well noted for the acquisition of RPS [27,28]. The TGFB1_509 T allele and the VEGFA _406 CC genotype are associated with a risk of renal scarring after UTI [29]. Polymorphism in the intercellular adhesion molecule 1 exon 4 was less common in patients who developed RPS in one study [30]. Polymorphisms of the angiotensin-converting enzyme and ANG II type 1 receptor genes are also implicated in the risk of RPS acquisition [31,32].

As observed already, although several significant mutations [22-32] have been identified in the innate immune response that may explain the risk of UTI or RPS, much is still to be discovered, and further research is needed to elucidate and validate host factors and genetic variations that may predispose to UTI or RPS [33]. In the quest to summarize the genetic host factors that predispose to RPS, a 2011 meta-analysis of cumulative studies showed only a modest association between RPS after UTI and the vasomotor genes involving the angiotensin-converting enzyme insertion or deletion polymorphisms and the inflammatory genes involving TGF-β1 c.−509 T>C polymorphisms [33]. Although heterogeneity among the studies was large, some gene expression differences were observed that could not be explained by differences in study design, and a few possible candidate genes have been investigated [33]. The role of inflammatory genes in RPS also warrants further investigation, as Hewitt et al also demonstrated that early treatment of APN in infants and young children had no significant effect on the incidence of subsequent renal scarring in an Italian cohort [34]. Bearing the foregoing in mind, apolipoprotein 1 genetic variant is suggested as the risk factor in the pathogenesis of RPS following febrile UTI.

African Americans have a three-fold higher lifetime risk of ESRD as compared with non-Hispanic whites because of a higher incidence of CKD and glomerulonephritis [35,36]. Recent studies also suggest that blacks living in sub-Saharan Africa (African blacks) may have a similarly high predilection to kidney disease as do African Americans, and these two populations (African Americans and African blacks) may share a common genetic predisposition [37,38]. Two apolipoprotein L1 (APOL1) susceptibility gene variants (G1 and G2) have been identified, with 88% higher risk of CKD progression in African Americans, and have also been suggested for the higher predilection of kidney disease in African blacks [39]. The APOL1 gene variants encode for circulating APOL1, which functions as a trypanolytic factor capable of killing the trypanosome parasites in the human serum [40-43]. People who have at least one copy of either the G1 or G2 variant are resistant to infection by trypanosomes, but people who have two copies of either variant are at an increased risk of developing nondiabetic kidney diseases, including hypertension-attributed end-stage kidney disease (ESKD), HIV-associated nephropathy (HIVAN), and focal segmental glomerulosclerosis (FSGS) [37,44]. The prevalence of the risk alleles in African Americans with these kidney diseases shown in recent studies are 67% in HIVAN, 47% in hypertension-attributed ESKD, and 66% in FSGS [37,44]. FSGS is a pattern of injury consisting of a sclerotic area made up of extracellular matrix and fibrous tissue; curiously, secondary FSGS can follow any injury that results in a substantial decrease in nephron number as seen in severe advanced primary tubulointerstitial diseases such as chronic obstruction or pyelonephritis [45].

Although possession of the APOL1 risk variants in an autosomal recessive manner increases susceptibility to nondiabetic kidney disease, not all people who possess these variants develop kidney disease, which indicates that another factor may initiate progression of kidney disease [45,46]. In addition, several studies suggest that one or both of the APOL1 risk variants may be gain-of-function mutations rather than loss-of-function mutations as the recessive mode of inheritance would suggest [46,47]. Thus, the kidney risk variants APOL1 may have acquired toxic properties rather than lost attributes essential for kidney health [47]. The APOL1 is a 398 amino acid protein with five functional domains, including the S domain-secretory signal, the membrane-addressing domain that serves as pH sensor and regulator of cell death, the BH3 domain associated with programmed cell death, the pore-forming domain, and the serum resistance-associated binding domain that confers resistance to trypanosoma brucei [48].

For the following reasons, APOL1 is hypothesized to play a role in the pathogenesis of RPS, as febrile UTI may be a second-hit insult for the APOL1 kidney disease risk variants:

1. APOL1 is resident in vascular endothelium, podocytes, proximal tubules, and arterial cells [49,50].
2. Two renal transplantation studies suggest that the APOL1 kidney risk allele association is mediated by the gene product isoform that is endogenously expressed within the kidney and not the circulating APOL1 [51,52].
3. APOL1 is a member of the family of BH3-only proteins that interacts with the family of Bcl2 proteins to help regulate their function in autophagy and apoptosis [53,54].
4. Although apoptosis is a beneficial process for the host in lower UTI as it results in exfoliation of the superficial cells of the multilayered epithelium and thus the eradication of the bacteria attached to and invaded into the cells, however, where the epithelium is single-layered and close to the underlying kidney tissue and blood vessels, apoptosis is more likely to be part of a deleterious cycle of tubular atrophy, cytolytic events, and renal scarring [55,56].
5. The expression of APOL1 in human embryonic umbilical vein endothelial cells can be induced by lipopolysaccharide [56] and by circulating inflammatory cytokines, including IFN-γ and TNFα [55], which supports the role of APOL1 in up-regulating the innate immune response to UTI.
6. APOL1 is involved in innate immunity, which is the primary response of the human host to bacterial invasion of the urinary tract or kidney tissue. It is up-regulated by proinflammatory cytokines gamma interferon and TNFα [57]. These molecules are known to attract neutrophils to the kidney during infection, and hence, the consequent RPS that may occur, as explained previously [15].
7. In cell culture, interferon and toll-like receptor agonists increased APOL1 expression by up to 200-fold; in some cases with the appearance of transcripts not detected under basal conditions. PolyI:C, a double-stranded RNA TLR3 agonist, increased APOL1 expression by up-regulating interferon directly or through an interferon-independent, IRF-3-dependent pathway [46].
8. The innate immune response to bacterial UTI also involves IRF-3 stimulating pathway, and there may be an increased APOL1 expression [46]. IRF3 _925 A/G and _776 C/T polymorphisms involved in neutrophil recruitment are strongly associated with febrile UTI susceptibility [46].
9. Little is still known about the roles of APOL1 in kidney disease. Over time, there has been an extension in the spectrum of APOL1-associated kidney diseases, including systemic lupus erythematosus [57,58], membranous nephropathy [59], sickle cell disease [60], and even an association of two risk variants of APOL1 in diabetic patients with CKD [61,39].

This study hypothesizes that APOL1 may be associated with RPS of UTI and that UTI is a trigger that determines RPS in susceptible individuals with APOL1 risk variants.

In addition to the limited availability of DMSA scanner, the issues of cost and exposure to radiation in children have prompted researchers to seek clinical or laboratory predictors of RPS. Most of the studies on the prevalence and clinical predictors of RPS following UTI were conducted in Europe, North America, Australia, and the Middle East and in Asia, with little or no data from sub-Saharan Africa, a region of the world where the APOL1 risk alleles are highly prevalent [62-73]. The researchers in these other continents have reported the risk factors for renal scarring in children with UTI to include age at diagnosis, gender, delayed treatment, recurrent infections, peak of fever, laboratory indices of inflammation (total white blood cell, WBC, count; procalcitonin, PCT; and C-reactive protein, CRP, concentration), the extent of renal parenchymal lesions, race or ethnicity, and the presence of VUR [62-73]. In fact, in a systematic review by Shaikh et al, VUR was found to be an independent risk factor for RPS [74], and VUR, notably, is also the most important risk factor for RPS following UTI [30]. Although VUR is rare in black children compared with their white counterparts [75], blacks are known to be more prone to kidney damage [37,38]; so it is possible that this predisposition is by far more potent and basically removes the advantages conferred by the lower rates of VUR. In addition, Ataei et al [76] and Benador et al [77] did not find age less than 5 years to be a predictor of RPS in their studies, and other authors also did not find any risk factor predictive of RPS in their studies [78,79]. Furthermore, in a meta-analysis by Faust et al, RPS was reported more among children in Asia than in the Middle East or in Australia [80], indicating a racial predisposition to RPS.

In view of the conflicting reports regarding the predictors of RPS among children with UTI, carrying out a well-designed, highly powered prospective cohort study to determine the risk of acquiring RPS in Nigerian children with a febrile UTI will contribute to the existing data of RPS in children with UTI. The findings of this study will for the first time provide information on the susceptibility factors ethnic sub-Saharan Africans may have for the development of RPS following a febrile UTI.

Africa, and in fact Nigeria, has complex populations and variations in climate, diet, and exposure to infectious diseases, which makes it a good setting for studying diversities in human genetics and phenotypes [81]. In this way, this work proposes to study the effects of APOL1 kidney risk variants on the acquisition of RPS following a febrile UTI in Nigerian under-five children. A better understanding of the risk factors for RPS following a febrile UTI would allow for individualized care models that would allow for a better prediction of RPS.

The overarching hypotheses are that RPS is a common complication of a febrile UTI in Nigerian under-fives and that the possession of APOL1 kidney risk variants increases the risk of RPS in these children. The study also proposes that clinical and laboratory predictors of RPS exist among children presenting with a first febrile UTI.

### Specific Aims and Hypotheses

#### Specific Aim 1: To Determine the Prevalence of Urinary Tract Infection Among Febrile Under-Five Children Attending the Emergency Pediatric Unit of the National Hospital, Abuja, Nigeria

Hypothesis 1: UTI is common among Nigerian under-fives with fever, with or without a specific source of the fever.

The presence of UTI among Nigerian children aged between >1 month and 60 months, presenting with axillary temperature ≥37.5˚C at the emergency pediatric unit (EPU) shall be determined. Children less than 1 month of age shall be excluded because their clinical presentations are unique. This is because they may present with hypothermia instead of fever [82]. There is also the need for immediate empiric antibiotics for the febrile neonate [82]. Also to be excluded are children who have been treated for UTI, children who have been on antibiotics 2 weeks before presentation, and children with known a neurological lesion causing bladder dysfunction, or those with a known stone disease. Urine collection will occur before the commencement of antibiotics if indicated for the presenting fever, as a single dose of an effective antibiotic rapidly sterilizes the urine. A midstream, clean-catch specimen will be obtained from children who have urinary control. A properly labeled universal bottle will be used to collect the urine sample. Prior cleansing of the perineum or urethral orifice will be done. In the infant or child unable to void on request, the urine specimen for culture will be obtained by suprapubic aspiration or urethral catheterization, and the procedures will follow the standard sterile technique. Suprapubic aspiration will also be the method of choice for obtaining urine from uncircumcised boys with a redundant or tight foreskin, from girls with tight labial adhesions, and from children of either sex with clinically significant periurethral irritation [2]. When tests on the urine will not be performed within the first hour of urine collection, urine will be stored in the refrigerator (at 4˚C) and will be tested within 4 hours of storage in the refrigerator [8,83]. Urine refrigerated will be kept at room temperature for 15 min before tests will be performed on them [83]. The urine so collected will be divided into two equal parts—one part for urine culture and the other part for dipstick urinalysis, enhanced urinalysis, and automated urinalysis. UTI will be defined as a positive test result for pyuria by either microscopy (≥5 WBCs per high-power field, [HPF] in uncentrifuged urine specimen) or dipstick test (positive leucocyte esterase test) and a positive growth on culture of at least 50,000 colony-forming unit (CFU) per mL of a single uropathogen in urine specimen obtained by catheterization or greater than 100,000 CFU per mL of a single uropathogen in clean-catch urine specimen or any uropathogen growth in urine obtained suprapubically [84]. Urine confirmation for UTI shall be done at the microbiology laboratory of the National Hospital, Abuja, and urinary inoculation on culture media and interpretation shall be done by a consultant microbiologist. Antibiotic treatment will be started empirically for all children as soon as possible based on epidemiology data at the National Hospital and, if required, changed to appropriate antibiotics according to the results of the sensitivity tests. A study proforma will be used to obtain the following information from the subjects: (1) sociodemographic data: age, gender, place of residence (urban or rural), ethnicity or tribe, and socioeconomic status of the household; (2) past medical history: prior use of antibiotics, past history of UTI, family history of recurrent UTI, family or subjects’ history of congenital anomaly of the urogenital tract (ie, VUR), prior history of worm infestations, history of constipation, and history of breastfeeding in the first 6 postnatal months; (3) symptom: jaundice, poor feeding, vomiting, diarrhea, irritability, strong smelling urine, abdominal pain, flank or back pain, irritability, dysuria, frequency, dribbling, poor stream, or straining to void; (4) signs: acutely ill-looking or not, degree of fever (≥37.5-38.5, >38.5-38.9, ≥39) and duration of fever before presentation (0-5 days, 6-14 days, >14 days), undernutrition as determined from anthropometric (height or length, weight, midarm circumference, occipitofrontal circumference) measurement, tenderness of the flank or costovertebral angle, suprapubic tenderness, abdominal tenderness, circumcision, signs of irritation on the external genitalia, pinworms, vaginitis, trauma, or sexual abuse; and (5) comorbidity: malaria, HIV, sickle cell disease, sepsis, upper respiratory tract infection or otitis media, pneumonia, nephrotic syndrome (NS), viral exanthema, malignancies, etc.

#### Specific Aim 2: To Determine the Prevalence of Renal Parenchymal Scarring Among Febrile Under-Five Nigerian Children With a Confirmed Urinary Tract Infection

Hypothesis 2: RPS is a common complication of UTI among febrile under-five Nigerian children.

RPS shall be determined with technetium Tc 99m DMSA renal scintigraphy. However, as studies have demonstrated that many abnormalities seen in DMSA scan done at 2 weeks resolve over time, and that little benefit exists in doing DMSA scan at 2 weeks, DMSA scan will be performed 6 months after the treatment for UTI to confirm or rule out chronic parenchymal scarring [8,85,86]. The DMSA scanning will be done at the National Hospital, Abuja, under the supervision of a consultant radionuclide physician. A kidney without uptake defect and 45% or greater relative (split) function will be classified as normal (DMSA class 0), and a kidney with decreased or absent uptake in one or more areas, or relative function less than 45% will be considered abnormal. The extent of kidney damage will be graded arbitrarily as class 1—uptake defect with 45% or greater relative function, class 2—40% to 44% relative function, and class 3—less than 40% relative function. In cases of bilateral renal damage, the kidneys will be individually classified by uptake defect extent. In cases of unilateral duplication, the expected mean normal split function will shift from 50% to 54%. Thus, the lower limit of normality will be considered at 49% [87].

#### Specific Aim 3: To Perform Genotyping and Analysis of Known Disease Susceptible Variants in the Apolipoprotein L1 Gene in Children With Renal Parenchymal Scarring Following Febrile Urinary Tract Infection

Hypothesis 3: APOL1 nephropathy risk variants G1 and/or G2 are associated with RPS following a febrile UTI.

For DNA samples, blood samples will be obtained from all children with confirmed UTI. We shall collect about 1.5 mL of whole venous blood. DNA will be extracted using labeled collection tubes for blood. All samples will be allocated a unique identifier and will be stored at −4˚C. The blood samples shall be shipped to a reputable diagnostic molecular laboratory in London, United Kingdom. The new *Axion Genome-Wide Pan-African Array Set* will be used for the genome-wide genotyping. At the least, three specific APOL1 candidate single nucleotide polymorphisms (SNPs) will be genotyped, including rs73885319 and rs60910145 in G1 and rs71785313 in G2 [51]. Cases would then be children with no VUR who develop RPS at 6 months following UTI, and controls will be children with confirmed UTI but without RPS scarring at 6 months.

#### Specific Aim 4: To Determine the Association Between Vesicoureteral Reflux and Febrile Urinary Tract Infection

Hypothesis 4: VUR is common in Nigerian under-fives with febrile UTI.

Micturating cystourethrogram (MCUG) shall be performed at 2 weeks of follow-up on children with confirmed UTI who also have abnormal renal and bladder ultrasound (RBUS) features, including hydronephrosis, scarring, high-grade VUR, or obstructive uropathy, in line with the 2011 American Academy of Pediatrics Clinical Practice Guideline that took into consideration the fact that MCUG is an uncomfortable, costly procedure that involves exposure to radiation [8]. The RBUS shall be done routinely at the first contact for all children with confirmed UTI.

At this time point (2 weeks), the MCUG will be done when the child must have received the full course of antibiotics treatment for the UTI. VUR will be graded into five classes as follows [88]: grade I—only fills the ureter but no dilation; grade II—fills ureter, pelvis, and calyces but without dilation and normal appearing calyces; grade III—mild or moderate dilation of the ureter and pelvis but no or only slight blunting of the fornices; grade IV—moderate dilation or tortuosity of the ureter with mild dilation of renal pelvis and calyces and blunting of the calyces; and grade V—gross dilation and tortuosity of the ureter, gross dilation of renal pelvis and calyces, and papillary impressions are no longer visible in most calyces.

The RBUS and the MCUG will be done by a consultant radiologist at the radiological department of the National Hospital, Abuja.

#### Specific Aim 5: To Assess the Association Between Predictor Variables Assessed at the Time of the Febrile Urinary Tract Infection and the Development of Renal Parenchymal Scarring at 6 Months of Follow-Up

Hypothesis 5a: Clinical and laboratory variables exist that can predict the risk of RPS at 6 months following a febrile UTI.

Hypothesis 5b: Serum TNF-α is more sensitive and specific than IFN-γ, PCT, CRP, erythrocyte sedimentation rate (ESR), and polymorphonuclear cell count in predicting renal RPS following a febrile UTI.

A comprehensive data of the sociodemographic, clinical signs and symptoms, examination findings, and the presumptive diagnoses will be collected on the first encounter for each child after informed consent has been obtained from the parents or caregivers of the children. About 2 mL of blood will be collected for complete blood counts, ESR, CRP, PCT, TNF-α, and IFN-γ. Other routine investigations will be as for the diagnostic work-up toward the presumptive diagnosis. The following factors will be considered for inclusion in the prediction model for RPS: age, sex, ethnicity, measured temperature at the time of diagnosis, duration of fever before presentation, grade of VUR, the organism isolated from culture (*Escherichia coli* vs other), results of renal ultrasonography (normal vs any abnormality), and levels of inflammatory markers (CRP, ESR, TNF-α, IFN-γ, PCT, and polymorphonuclear cells).

Elevated acute phase reactants will be taken as follows: CRP >40 mg/L [89], ESR >20 mm/hour [90], PCT *≥* 0.5 ng/mL [91], and polymorphonuclear cells count >60% [92].

PCT is produced by the liver and peripheral blood mononuclear cells and modulated by lipopolysaccharides and sepsis-related cytokines. PCT is very specific for bacterial infection and helps to distinguish between viral and bacterial infections, which are particularly useful in children [93].

Although TNF-α and IFN-γ are both immunomodulating and proinflammatory cytokines, TNF-α secretion by activated macrophages is via bacterial lipopolysaccharide, whereas, IFN-γ is critical for innate and adaptive immunity against viral, some bacterial, and protozoal infections [94,95]. Thus, this study proposes that TNF-α may be more specific and sensitive than IFN-γ and other proinflammatory proteins in detecting the host’s inflammatory response to invading bacterial UTI and hence, the consequent RPS.

### Expected Impact of Study

This study is important because it is coming at a time when genetic studies have started to explain the differences in the predisposition to kidney diseases in blacks compared with white. The study seeks to unravel for the first time the potential role of APOL1 kidney risk alleles in the evolution of RPS consequent on febrile UTI. The prevention of RPS is the most important goal of all therapies for childhood UTI, and if APOL1 kidney risk variants are shown to be associated with RPS, it will offer less invasive genetic biomarkers for predicting children who will develop RPS following UTI. This approach will reduce the need for urinary tract imaging that is expensive and exposes children to toxic radiation. This study will also offer an opportunity for clinical and translational research of kidney disease, as it will expose the researcher to training in genomic science and genetic studies.

### Significance of Study

The significance of study is as follows:

1. Although children with UTI tend to present with fever, it is often difficult on clinical grounds to distinguish UTI from other febrile illness. This makes UTI one of the most often missed diagnoses in the pediatric wards in developing countries [96,97]. UTI, whether confirmed or undiagnosed, has greater significance in childhood than in adults as most renal scars occur after such infections in the first 5 years of life [6-8,98]. The finding of this study would add to the body of evidence, suggesting that all children aged less than 5 years presenting with fever at the EPU be screened for UTI.
2. The long-term complications of febrile UTI (APN) have been previously studied, and they include the risk of RPS, hypertension, preeclampsia, and ESKD that may ultimately require dialysis or renal transplantation [99]. These long-term complications have been linked with the evolution of RPS. This will be the first comprehensive study that would determine the clinical and laboratory risk factors of RPS acquisition following a febrile UTI in African children, resident in Nigeria. The study may produce an easily implementable clinical and laboratory prediction models that could be used to identify children at risk for renal scarring. Furthermore, if the risk of developing RPS following a febrile UTI is sufficiently high, it would make necessary the request for DMSA scan a worthwhile and cost-effective diagnostic follow-up in a low-income country such as Nigeria.
3. Some researchers did not find any clinical predictors of RPS following UTI [77,78]; a recent meta-analysis by Shaikh et al in 2014 also could not make a conclusive recommendation for the use of PCT, CRP, and ESR [65] in predicting APN from cystitis because of paucity of studies. The target population of the present research will therefore add to the pool of information that seeks to determine the potential role of acute phase reactants, including ESR, CRP, PCT, TNF-α, and IFN-γ in predicting the risk of renal parenchymal involvement and subsequent RPS in febrile children with UTI. In particular, the research would seek to determine the role of TNF-α and IFN-γ in predicting RPS, as these proinflammatory cytokines are known to up-regulate the expression of APOL1 in the kidney tissues [55,56].
4. RPS following UTI is an important cause of renal morbidity in children. Studies have shown that the intensity of the inflammatory response following infection is related to the risk of RPS. However, genetic variability in this response has not been well studied [30]. This study proposes propose that possession of G1 and/or G2 APOL1 kidney risk alleles pose a “gain-of injury” in the evolution of RPS following a febrile UTI. If APOL1 risk variants are found to be associated with RPS, it would make a strong case of assaying for APOL1 risk variants as a genetic biomarker for RPS among febrile black African under-five children with UTI. It would eliminate the need for exposure to expensive DMSA irradiation for diagnosing RPS.
5. VUR is one of the most common inherited diseases of the genitourinary tract in children. The incidence of primary VUR is 1% in normal infants, whereas it is 30% in infants presenting with UTI [100]. VUR notably is also the most important risk factor for RPS following pyelonephritis [30]. However, in a Nigerian 5-year prospective study that involved 699 patients with renal disorders, although UTI accounted for most of the renal disorders (68.9%), no child was found with VUR [101]. This study will add to the pool of data seeking to determine the contribution of VUR to UTI and subsequent RPS in Nigerian children.
6. Presently, there is a paucity of epidemiologic, genomics, and translational studies of kidney disease among Africans. There is inadequate knowledge and exposure to genetic and translational longitudinal studies. The future of predictive, preventive, and individualized medicine in Africa is therefore gloomy but could be remedied by training more clinician researchers. Hopefully, there is a lot to be gained by the researcher in terms of training and exposure in the process of executing this research work.

The researcher’s capacity to conduct this study is presented in Multimedia Appendix 1.

## Methods

### Experimental Design

This is a prospective, observational, longitudinal, case-control cohort study involving febrile under-five children presenting at the EPU of the National Hospital, Abuja. The conceptual framework is as depicted in Multimedia Appendix 2. The initial framework was originally from the schematic illustration of the pathogenesis of UTI by Johnson et al [102].

#### Study Environment

The National Hospital is located in Abuja, the Federal Capital Territory of the Federal Republic of Nigeria. Abuja is located in the North Central Geo-Political Zone of Nigeria. It occupies a land area of 7753.9 square kilometers [103]. Abuja grew by 139.7% from 2000 to 2010, making it the fastest growing city in the world [104].

#### Target Population

As of 2016, Abuja’s population is estimated at 6 million persons [105]. Abuja is a newly created city where all Nigeria’s 250 ethnic tribes are expected to live together in harmony. The indigenous inhabitants of Abuja are the Gbagyis, the Bassas, the Gwandaras, the Gedes, the Ganaganas, and the Koros; however, the major tribes of Hausa, Yoruba, and Igbo also reside in the city in large numbers. All the inhabitants of Abuja are expected to patronize the National Hospital, situated at the Independence Avenue, Phase 2, Abuja. The city would, therefore, provide a good repository of diverse genetic representation suitable for testing the influence of APOL1 kidney risk variants on RPS acquisition. The National Hospital was established under Decree 36 of 1999 but was commissioned on May 22, 1999 [106]. It is a tertiary health institution that prides itself as a tertiary health facility with a state-of-the-art technology in a conducive and clean environment. It is the only hospital in the whole of Northern Nigeria that has the radionuclide scanning machine, hence, the suitability for this study that seeks to determine the evolution of RPS following febrile UTI. In 2016, the hospital had a pediatric outpatient attendance of 1575, with an average of 132 children per month [107].

#### Study Population and Eligibility Criteria

The intention is to enroll a minimum sample size of 500 consecutive febrile under-five children, whose parents or caregivers consent to the study’s objectives. This sample size was expanded from the 260 derived from the Leslie Kish formula [108] at a standard normal deviation of 1.96 (corresponding to 0.5% CI), with a degree of accuracy set at 0.05 and using the prevalence of 21.4% for UTI among febrile under-five children in a similar study by Adedoyin et al [12].

However, to test the study’s hypotheses, cases will be children with a confirmed RPS following a febrile UTI, and an equal number of controls (age-, gender-, and ethnicity-matched children with a febrile UTI but without RPS) will be targeted from the sample size. The study would aim at having at least 50 cases and 50 controls.

The inclusion criteria are shown in Textbox 1. The exclusion criteria are shown in Textbox 2.

#### Recruitment

Four research assistants (RAs) will be employed for the purpose of recruiting the participants for this study. This will comprise 2 medical doctors and 2 nurses. The medical doctors shall be involved in conducting the dipstick urinalysis, automated urinalysis, anthropometrical measurements, and the collection of urine and blood samples from the recruited participants. The nurses will be in charge of obtaining informed consent, the administration of the questionnaire, and the follow-up of the recruited subjects to the radiological department of the National Hospital, Abuja. The RAs will be trained on the concept and objectives of the study and the quality and culture appropriateness of the study’s questionnaire will be discussed with them. The proficiency of the RAs will be verified via role-playing. The study will use brochures, posters, flyers, newsletters, and family engagement to promote recruitment and retention within the study cohort. Parents or caregivers of febrile children coming to the EPU will be approached to be screened for enrollment. Furthermore, the study shall engage the services of a microbiologist, a radiologist, and a radionuclide physician. The microbiologist will be responsible for doing urine culture and interpretation, urine microscopy, and enhanced urinalysis. The radiologist shall be responsible for doing the RBUS and the MCUG. The radionuclide physician shall be responsible for the DMSA scanning for detecting APN and RPS.

Financial compensation will be made available for parents or caregivers who would have to bring their infants back to the National Hospital for MCUG at 14 days and for the DMSA scan at 6 months, following the treatment for the febrile UTI. To this end, the mobile telephone numbers of the consenting parents or caregivers of the children will be obtained for tracking after discharge from the hospital. In the same way, extra efforts will be made to obtain traceable home addresses of the study participants. Attrition would be minimized by ensuring that parents or caregivers fully understand this responsibility or expectation of study’s protocol at the time of signing the consent forms.

#### Obtaining Ethical Access

Ethical approval for the study shall be obtained from the research and ethics committee of the National Hospital, Abuja and the institutional review board of the university that will be willing to supervise this proposal as a PhD thesis. A written informed consent (see Multimedia Appendix 3) shall be obtained from parents or caregivers of all children that will make the sample population of this study. The confidentiality and the privacy of research participants will be protected. The details of the informed consenting are as contained in Step 1 under the enrollment below. A rapid direct data sharing with the research community will be in accordance with the National Institutes of Health data sharing policy.

**Inclusion criteria.**

- Consecutive febrile children (≥1 month-59 months of age) presenting at the emergency pediatric unit (EPU) of the National Hospital, Abuja, whose parents or caregivers give consent to the study.
- Fever is defined as axillary temperature ≥37.5˚C [109] using the mercury thermometer.
- Urinary tract infection (UTI) will be defined as a positive test result for pyuria by either microscopy (≥5 white blood cells per high-power field in uncentrifuged urine specimen) or dipstick test (positive leucocyte esterase test) and a positive growth on culture of at least 50,000 colony-forming unit (CFU) per mL of a single uropathogen in urine specimen obtained by catheterization or greater than 100,000 CFU per mL of a single uropathogen in clean-catch urine specimen or any uropathogen growth in urine obtained suprapubically [84].
- Renal parenchymal scarring (RPS) will be defined as a kidney with a decreased or an absent uptake in one or more areas, or relative function less than 45% on a dimercaptosuccinic acid (DMSA) scan done at 6 months following a confirmed febrile UTI.
- Parents or caregivers’ ability to understand and comply with planned study protocols.
- Parents or caregivers’ ability to provide informed consent before recruitment into the study.
- Parents or caregivers residing in study area and at least within 5 miles radius, for easy follow-up and to reduce the number that may be lost to follow-up.

**Exclusion criteria.**

- Febrile under-five children who had taken antibiotics in the preceding 2 weeks.
- Children who have been treated for urinary tract infection (UTI) before.
- Children with neurological lesion causing bladder dysfunction or those with known stone disease
- Children with known congenital abnormalities of the kidney and the urinary tract (CAKUT, ie, vesicoureteral reflux, VUR; cystic kidney diseases; renal dysplasia; renal hypoplasia).
- Children with HIV or AIDS and those with sickle cell anemia, as these diseases may confound the finding of renal parenchymal scarring (RPS).
- Children with mixed ethnicity defined as having more than one ethnic ancestry in the biological parents and grandparents.
- Children who are already part of another ongoing research effort.
- Children whose parents or caregivers will refuse to give informed consent for participating in the study.
- Children whose parents or caregivers are institutionalized (eg, prisoner, nursing home residents, and prisons).

#### Step-by-Step Recruitment Process for the Study

##### Step 1: Enrollment

The first step is to do screening to confirm study eligibility and provide participants with information about the study. A questionnaire (see Multimedia Appendix 4 for the sample of the study’s questionnaire) assessing eligibility will be completed, and contact information, including mobile phone numbers of the parents or caregivers, their relatives, or neighbors will be obtained. Traceable home addresses of the participants will also be obtained. Informed consent will then be obtained from the parents or caregivers of the prospective study’s participants. Consenting will be done in the privacy of the consultation room (isolated from ambient noise and distractions) where the participant is being attended to. Consenting will be in simple understandable English. When necessary, translations into the participants’ native language will be done for a better understanding. Before signing the informed consent, the details of the consent form shall be orally reviewed with the potential participant and answers to any questions that the participant has concerning participation in the study shall be given. The original signed consent form will be stored in the participant’s study file, and a copy of the signed consent form will be given to the participant. Specifically, the following must be accomplished during the informed consent process:

1. The participant will be informed that participation in the study is *voluntary* and that refusal to participate will involve no penalty or loss of benefits or negative impact on their medical care.
2. The participant will be informed of the *purpose* of the study and that it involves *research*.
3. The participant will be informed of *any alternative procedures*, if applicable.
4. The participant will be informed of any foreseeable *risks*.
5. The participant will be informed of any *benefits* from the research.
6. An outline of safeguards to protect participant *confidentiality* will be included, as well as an indication of the participant’s right to withdraw without penalty. This would be balanced with a discussion of the effects withdrawals have on the study and the responsibility a participant has, within limits, to continue in the study if he or she decides to enroll.
7. The participant will be informed *whom to contact* for information about research subjects’ rights, information about the research study, and in the event of research-related injury.
8. The participant will be informed as to whether or not *compensation* is offered for participation in the study and/or in the event of a medical injury.
9. The participant will be informed that he or she will be notified of any significant changes in the protocol that might affect their willingness to continue in the study.
10. The informed consent form will be duly signed or thumb-printed and dated by the participant or witness before initiation of any study-related activity.

##### Step 2: Obtaining the Enrollees’ Sociodemographic Data, Other Relevant Information, Biological Specimens, and Renal and Bladder Ultrasound Scanning

A well-structured questionnaire (Multimedia Appendix 4) will be used to capture information relating to sociodemographics and other relevant information that may confound the outcome (UTI). It will take approximately 30 to 45 min to complete the questionnaire. The information the questionnaire seeks to obtain shall include the following:

1. Sociodemographic data: age as at last birthday, gender, place of residence (urban or rural), ethnicity or tribe, and socioeconomic status of the household
2. Past medical history: prior use of antibiotics, past history of UTI, family history of recurrent UTI, family or subjects’ history of congenital anomaly of the urogenital tract (ie, VUR), prior history of worm infestations, history of constipation, and history of breastfeeding in the first 6 postnatal months
3. Symptoms: jaundice, poor feeding, vomiting, diarrhea, irritability, strong smelling urine, abdominal pain, flank or back pain, irritability, dysuria, frequency, dribbling, poor stream, or straining to void
4. Signs: acutely ill-looking or not, fever (degree and duration before presentation), undernutrition as determined from anthropometric (height or length, weight, midarm circumference, occipitofrontal circumference) measurement, tenderness of the flank or costovertebral angle, suprapubic tenderness, abdominal tenderness, circumcision, signs of irritation on the external genitalia, pinworms, vaginitis, trauma, or sexual abuse
5. Comorbidity: malaria, sepsis, upper respiratory tract infection or otitis media, pneumonia, NS, viral exanthema, malignancies, etc

The following biological specimen will be collected from the enrolled child:

1. The urine sample will be collected as per the method relevant to the age of the child. Urine will be studied for culture and sensitivity and antimicrobial activity analysis. Furthermore, the urinalysis will be done by dipstick, urine microscopy, automated urinalysis, and enhanced urinalysis.
2. About 2 mL of blood will be collected using the sterile procedure for each child and sent for complete blood count, polymorphonuclear cell counts, ESR, CRP, PCT, TNF-α, IFN-γ, HIV, and hemoglobin genotypes.
3. Other laboratory work, including viral screening (hepatitis B or C), blood culture, stool microscopy, culture and sensitivity, cerebrospinal fluid culture and sensitivity, x-ray investigations, and joint fluid aspirate studies will be according to the diagnostic work-up for the particular child.
4. Additional 1.5 mL of blood shall be collected for APOL1 DNA analysis for all children with confirmed UTI.

RBUS scan will be done for all children with a confirmed UTI.

##### Step 3: A 14-Day Follow-Up of Children With Confirmed Urinary Tract Infection for Micturating Cystourethrogram

Any child with a confirmed febrile UTI as discussed previously will be subjected to MCUG at 14 days follow-up, *only if* the RBUS shows features of hydronephrosis, scarring, high-grade VUR, or obstructive uropathy. The MCUG would then confirm VUR and its grade. If it happens that the child has been discharged before this time, the parents or caregivers shall be contacted to bring the child for MCUG. Before this time point (2 weeks), RAs will be in constant contact with the parents or caregivers of discharged children via telephone calls and SMS text messages (short message service, SMS).

##### Step 4: A 6-Month Follow-Up of Children With Confirmed Urinary Tract Infection

All children with a confirmed UTI but without a VUR are expected to have a DMSA scan at 6 months of follow-up for evaluation of chronic RPS. Before this time point (6 months), the RAs will also be in constant contact with the parents or caregivers of discharged children via telephone calls and SMS text messages. The flowchart of the four steps is summarized in Multimedia Appendix 5.

#### Laboratory Procedures and Measurements

##### Serum Analysis

A total of 3.5 mL will be collected from the recruited subjects in step 2. The blood collected will be allowed to clot and centrifuged to produce the serum for biochemical analysis that will include serum TNF-α, IFN-γ, PCT, and CRP. The WBC counts and the differentials (polymorphonuclear cells) will be measured using the automated hematological analyzer (SYSMEX automated hematology analyzer KX-21N, Sysmex Corporation, Kobe, Japan). Serum levels of TNF-α and IFN-γ will be assayed by ELISA (BD Biosciences, United States) according to the manufacturer’s instructions. The TNF-α concentration will be calculated in the test samples on the basis of the curve produced by plotting the optical density values of the known standards (range: 7.5-500 pg/mL) on log-log graph paper.

The level of serum IFN-γ will also be detected with the IFN-γ ELISA kit. The detection limits of the kit for TNF-α and IFN-γ will be 2 and 1 pg/mL, respectively.

PCT will be measured with a quantitative immunoluminometric assay (LUMItest PCT, progressively replaced by PCT sensitive KRYPTOR, both from Brahms Diagnostica, Berlin, Germany), with a maximum interassay variation of approximately 0.3 ng/mL. CRP will be measured using the latex agglutination method and the automated method on Roche Integra 400 with an analytical goal of ±10%.

##### DNA Analysis

This study will employ the new “Axiom Genome-Wide Pan-African Array Set” to perform the genome-wide genotyping for variants in the APOL1 gene rather than the limited candidates’ SNPs (ie, rs73885319 and rs60910145 in G1 and rs71785313 in G2). DNA samples will be prepared and brought to a concentration of 100 ng required per array in the *Axiom Genome-Wide Pan-African Array Set*. The manufacturer’s protocol for Axiom 2.0 Assay Manual Workflow will be used for sample processing and preparation for genotyping. The Axiom genotyping array data will be analyzed by use of Affymetrix Power Tools to perform quality control analysis and sample and/or SNP filtering before the downstream analysis. The DNA analysis will be done at a reputable laboratory.

##### Urine Culture

The urine culture will be done within 1 hour of collection, employing the quantitative method as described by Guttmann and Stokes [110]. Each uncentrifuged urine sample will be well mixed and inoculated unto plates of cystine lactose electrolyte deficient medium and blood agar as described by Urquhart and Gould [111] and incubated aerobically at 37˚C for 24 hours after which the colonies will be counted with a colony counter.

The bacterial isolates will be identified based on colony morphology characteristics, Gram stain reaction, and biochemical tests using standard techniques [112]. Antibiotic sensitivity pattern of the isolates will be determined by the disc diffusion method in accordance with the National Committee for Clinical Laboratory Standards [113]. The discs will be placed on the agar surface and incubated for 24 hours. After incubation, the diameter of the zone of inhibition will be measured and compared with a zone diameter interpretative chart to determine the sensitivity of the isolates to the antibiotics.

When tests on the urine will not be performed within the first hour of urine collection, urine will be stored in the refrigerator (at 4˚C) and will be tested within 4 hours of storage in the refrigerator [114]. Urine refrigerated will be kept at room temperature for 15 min before tests will be performed on them [114].

##### Dipstick Urine Analysis

The uncentrifuged urine sample will be used, and the procedure will follow a standard method. The analysis will specifically look for leukocyte esterase, nitrite, hematuria, and proteinuria.

##### Microscopic Urine Analysis

The microscopic analysis will take place after centrifuging of the urine and will follow the standard procedure [114]. The finding of >5 WBCs per HPF in a centrifuged urine specimen will be considered as pyuria. Bacteriuria is the finding of bacteria on urine microscopy.

##### Enhanced Urinalysis

Uncentrifuged urine will be drawn into a Neubauer hemocytometer via capillary action [115]. Pyuria will be assessed by counting WBCs on each side of the chamber, averaging the value and multiplying by 1.1 to obtain the number of WBCs per mm^3^. Two drops of uncentrifuged urine will be placed on a sterile slide within a standardized marked area of 1.5 cm diameter, air-dried, fixed, and Gram stained. Bacteriuria will be assessed as the average number of bacteria per 10 oil immersion fields; morphology and Gram-stained smear results would be reported.

##### Automated Urinalysis

Automated urinalysis [115] operates on the principle of flow cell digital image capture in combination with a trained neural network (Auto-Particle Recognition, APR software). Aspirated urine is hydrodynamically focused between two layers of suspending fluid (planar flow), forcing particles to orient in a single plane facing a microscope objective lens coupled to a digital camera. Five hundred fields of digital image are captured per specimen. APR software is programmed to recognize the size, shape, contrast, and texture of urine particles. APR classifies particles into 12 categories, including red blood cells, WBC, WBC clumps, hyaline casts, unclassified casts, squamous epithelial cells, nonsquamous epithelial cells, yeast, bacteria, unclassified crystals, mucus, and sperm. Representative images are then screened by a technician for accuracy and confirmed or adjusted accordingly. For this study, digital images will be stored for subsequent editing by a single experienced technician (DL). Dipstick results will be obtained by reflectance spectroscopy and included pH, specific gravity, nitrite, and leukocyte esterase. The automated WBC count is reported per HPF, and a conversion factor of 5.5 will be used to convert the automated WBC values reported per HPF to mm^3^.

##### Other Tests

Other investigations, including blood culture, joint aspirates for microbiology study, chest x-ray, stool microscopy culture, and sensitivity will be according to individual appropriateness.

##### Renal and Bladder Ultrasonography

RBUS will be requested for all bacteriologically confirmed cases of UTI while they are still on admission. All children will be examined in a warm room. Mothers will be requested to give water to the children to drink 1 to 1.5 hours before scanning. The abdominopelvic scan will be done using an Ultrasound SDD-3500 Plus, Japan 2005 scan machine with a 3-5 MHz curvilinear transducer. With the patient lying supine, the abdomen will be exposed, and gel will be applied. The transducer will be placed on the abdomen and gently moved laterally to the right and the left flanks from the midabdomen for the visualization of the right and the left kidney, respectively. The transducer will also be moved to the suprapubic region to localize the urinary bladder, especially when adequately distended with urine.

##### Voiding Cystourethrography

VCUG is after antibiotic therapy and will be done at 2 weeks for cases of confirmed UTI having hydronephrosis, scarring, high-grade VUR, or obstructive uropathy on RBUS. Children will be told to void (for those who can obey a command), after which, a preliminary coned down view of the bladder is taken. With the patient lying supine on the x-ray table and under aseptic technique, a lubricated catheter will be introduced into the bladder, and any residual urine will also be drained. A total of 150 mL of contrast medium (Urografin or Utravist) will be introduced into the urinary bladder until it is adequately distended. When the radiologist is convinced that the child will micturate or when the child shows the urge to micturate, the catheter will then be removed quickly. Spot films will then be taken during micturition. Films of the entire urethra and a full-length view of the abdomen will also be taken to demonstrate any reflux into these organs.

##### Dimercaptosuccinic Acid Scan

The static renal scintigraphy will be done 2 to 4 hours after DMSA injection at a dose of 1 MBq/kg body weight (minimum 15 MBq). Planar images will be obtained by a high-resolution collimator in one posterior and two oblique projections with 300,000 counts in the posterior view. All data files will be evaluated by the consultant nuclear medicine specialist.

The maximum irradiation dose would be 2 millisieverts (I millisievert from MCUG and 1 millisiervert from DMSA scan [116])

##### Specimen Management

All specimens will be properly labeled and secured in a specimen bag with an accompanying laboratory request form. All specimens will be secured in a specimen carrier and transported to the study laboratory. All specimens will be delivered to the laboratory no longer than 3 hours after collection. Subject enrollment will occur only during weekdays and between 8.00 AM to 4 PM for logistic reasons. Specimens will be duly processed, and preliminary culture results will be made available to the researcher and the attending physician during the first 48 hours after enrollment and final results within 5 days. All clinical samples will be carefully labeled and coded using freezer-resistant labels. An electronic storage file will be developed to facilitate storage and retrieval of specimens.

- 2 mL of blood will be allowed to clot for 30 min, centrifuge for 20 min at 2400 rpm, and store at 4˚C and ship with cold packs for TNF-α AND IFN-γ assay.
- 1.5 mL of blood, stored at 4˚C and ship with cold packs for DNA analysis.
- Whenever there is a requirement to store sample at −20˚C or −80˚C, the freezer at the Professor Obaro’s Research Laboratory of the International Foundation Against Infectious Disease in Nigeria (IFAIN) will be used.
- A database will be created for logging all stored samples.

### Data Management

All participants will have a study identification to be used on study’s questionnaire and biospecimens. Questionnaires will be kept without patients’ identification information in a secured locked location as per the policies of Institutional Research and Ethics Board per site. All data will be checked for consistency, and outliers will be identified by examining empirical distributions of each outcome All data will be entered into REDCap, a secured online tool.

### Statistical Analysis

Statistical analysis will be done with SPSS version 21 (IBM Corp).

#### Statistical Analysis for Specific Aim 1

For numerical characteristics with symmetrical distribution, means and SD will be used as a measure of dispersion, whereas median and the interquartile range will be used to measure the central tendency for skewed numerical data. For categorical variables, proportions and percentage distribution would be described. Age grouping (≥1 month-2 months, >2 months-12 months, >12 months-24 months, >24 months- 59 months) will be done to take care of the known age-dependent risk of UTI [2]. Prevalence rates with 95% CIs will be calculated for UTI. Proportion distribution of UTI across the comorbidities will be described. The screening (ie, sensitivity, specificity, negative and positive predictive values) values of nitrite, leucocyte esterase, proteinuria, and hematuria on dipstick urinalysis will be compared with the urine culture. Furthermore, pyuria and bacteriuria identified on microscopic analysis and on enhanced urinalysis will be compared with bacteriologically confirmed UTI for sensitivity, specificity, and negative and positive predictive values. The dipstick urinalysis will be compared with the automated urinalysis in terms of screening values for UTI as confirmed by the urine culture. For children with a confirmed UTI, comparisons will be made between potential predictor variables (ie, sociodemographic variables, past medical history, symptoms and signs, and comorbidities) and UTI using chi-square test of proportions or, in the case of small samples, Fisher exact test. Predictors that attained significance at *P* value of ˂.05 (except a priori predictors such as age and grade of fever, regardless of the *P* values) will be considered for multiple logistic regressions to evaluate the possibility of confounding in the relationship with UTI. For all analyses, *P* values less than .05 will be considered statistically significant.

#### Statistical Analysis for Specific Aim 2

The prevalence rates of children who developed RPS will be calculated. Cases shall be children who develop RPS consequent to febrile UTI and who also do not have VUR. Controls shall be children with confirmed UTI but who do not develop RPS. The risk of developing RPS across the age groups (≥1 month-2 months, >2 months-12 months, >12 months-24 months, >24 months-59 months) will be determined. Proportion distribution of RPS in children with UTI will also be done across the various potential risk factors of RPS, including age at diagnosis of UTI, gender, delayed treatment (>6 days of fever), peak of fever, laboratory indices of inflammation (total WBC count, ESR, IFN-γ, TNFα, PCT, and CRP concentration), the extent of renal parenchymal lesions, ethnicity (the major Nigerian ethnic groups of the Yorubas, the Ibos, the Fulanis, the Hausas, and others categorized as Others), and the presence of VUR. Ethnicity of the child will be that of his biological father and mother and the grandparents. Chi-square test of proportions or, in the case of small samples, Fisher exact test will be used to test the association between each of these risk factors and RPS. Predictors that attained significance at *P* value of ˂.05 will be considered for multiple logistic regressions to evaluate the possibility of confounding in the relationship with RPS. For all analyses, *P* values less than .05 will be considered statistically significant.

#### Statistical Analysis for Specific Aim 3

The dependent variable is RPS and the independent variables will be the possession of either the G1 and/or the G2 APOL1 nephropathy risk variants. The possession of either the G1 and/or the G2 APOL1 will also be done among the cases defined by their ethnic groups of Yorubas, Ibos, Fulanis, Hausas, and Others. Ethnicity of the child will be that of his biological father and mother and the grandparents. Chi-square test of proportions or, in the case of small samples, Fisher exact test will be used to test the association between the possession of either the G1 and/or the G2 APOL1 risk variants and RPS. The APOL1 gene alleles associated with RPS will be described. Comparisons will be made between potential predictor variables and RPS using chi-square test of proportions or, in the case of small samples, Fisher exact test. At the least, the following factors will be considered for inclusion in the prediction model: age groups, sex, ethnicity, APOL1 gene allele, measured temperature at the time of diagnosis, duration of fever before presentation, the organism isolated from culture (*E coli* vs others), results of renal ultrasonography (normal vs any abnormality), and levels of inflammatory markers (TNF-α and INF-γ, CRP, ESR, PCT, and polymorphonuclear cells). In most cases, potential predictors will be dichotomized into either “yes” or “no.” Predictors that attained significance at *P* value of ˂.05 will be considered for multiple logistic regressions to evaluate the possibility of confounding in the relationship with RPS.

#### Statistical Analysis for Specific Aim 4

The outcome variable will be UTI, and the independent variables will be the various grades (I-V) of VUR. The effect of compounding with other significant risk factors (*P* value of ˂.05) for UTI derivable from Specific Aim 1 above will also be determined using the multiple logistic regression analysis.

#### Statistical Analysis for Specific Aim 5

Comparisons will be made between clinical predictor variables (ie, age, gender, ethnicity, past medical history, symptoms and signs, and comorbidities) and RPS using chi-square test of proportions or, in the case of small samples, Fisher exact test. In most cases, potential predictors will be dichotomized into either “yes” or “no.” Predictors that attained significance at *P* value of ˂.05 will be considered for multiple logistic regressions to evaluate the possibility of confounding in the relationship with RPS. For laboratory predictors, the sensitivity, specificity, and negative and positive predictive values of serum TNF-α, INF-γ, PCT, CRP, ESR, and polymorphonuclear cell count in detecting RPS would be compared. For all analyses, *P* values less than .05 will be considered statistically significant.

#### Gene Analysis

All candidate gene analyses will be analyzed. Each of the SNPs to be examined will be analyzed individually with RPS. Logistic regression model will be used for RPS, and the likelihood ratio test for significance will be used. The overall test of genotypic association with two degrees of freedom and any statistical contrasts will be defined by three genetic models: dominant, additive, and recessive models, respectively (each with one degree of freedom), with and without adjustment for covariates. If the test of general association is significant, then three a priori genetic models will be explored, and the best genetic model will be selected without further adjustment for multiple comparisons. Multiple SNPs from each gene will be tested, and Bonferroni correction to account for multiple testing will be done. The effect size for each of the risk haplotypes will be tested using a generalized linear model defined depending on the outcome variable.

Table 1 is the summary of the statistical analyses of the primary and secondary outcomes of the study.

#### Power Calculations

The graphical representation of the power to detect association in the 500 minimum sample size populations can be seen in Multimedia Appendix 6.

For the proposed 500 minimum sample size, and assuming frequency of the exposure risk of haplotypes of APOL1 G2 (2 risk alleles) at 15% in the general population [117], the calculation indicates that the proposed sample size provides sufficient power (≥80%) to detect significant association, if present.

#### Timeline of Study

Table 2 depicts the timeline of the research proposal that is expected to last for 3 years.

**Table 1:** Statistical analyses of the primary and secondary outcomes of the study.

Outcome		Definition	Variable	Analyses
**Primary**				
	Urinary tract infection (UTI) among febrile children aged 1 month to 5 years	UTI will be defined as a positive test result for pyuria by either microscopy (≥5 white blood cells per high-power field in uncentrifuged urine specimen) or dipstick test (positive leucocyte esterase test) and a positive growth on culture of at least 50,000 colony-forming Unit per mL of a single uropathogen in urine specimen obtained by catheterization or greater than 100,000 CFU per mL of a single uropathogen in clean-catch urine specimen or any uropathogen growth in urine obtained suprapubically	Dichotomous (yes or no)	Prevalence ratios, odds ratio (OR), 95% CI
**Secondary**				
	Renal parenchymal scarring among febrile children aged 1 month to 5 years with confirmed UTI	A kidney with decreased or absent uptake in one or more areas or relative function less than 45% on dimercaptosuccinic acid scan	Dichotomous (yes or no)	Prevalence ratio, OR, 95% CI, logistic regression

**Table 2:** Timeline of study

Activity		Year 1	Year 2	Year 3
**Study year 1: 1-6 months**				
	Institutional review board approval	January to June		
	Hiring and training of research assistants	January to June		
	Engagement of microbiologist, radiologist, and radionuclide physician	January to June		
**Study year 1: 6-12 months**				
	Project enrollment	July to December		
	Shipping of samples for apolipoprotein L1 (APOL1) analysis	July to December		
**Study year 2: 1-12months**				
	Project enrollment		January to December	
	Shipping of samples for APOL1 analysis		January to December	
**Study year 3: 1-6 months**				
	Project enrollment			January to June
	Shipping of samples for APOL1 analysis			January to June
**Study year 3: 6-12 months**				
	Analysis of data			July to December
	Writing reports and manuscript			July to December
	Defense of PhD thesis			July to December

## Results

### Primary Outcome

The primary outcome measure is UTI in febrile children aged 1 month to 5 years.

### Secondary Outcome

The secondary outcome measure is RPS among the children with confirmed UTI. The study will show for the first time the burden of RPS following a febrile UTI among Nigerian under-five children. It will also highlight if the risk of acquiring RPS is dependent on having APOL1 kidney disease risk variants among the children with confirmed febrile UTI.

Participant recruitment for this case-control cohort study will commence when the researcher gets a placement for a PhD study and after securing adequate funding grants for the study. The study is expected to last for at least 3 years.

## Discussion

### Principal Findings

This study will employ the new *Axiom Genome-Wide Pan-African Array Set* to perform the genome-wide genotyping for variants in the APOL1 gene rather than the limited candidates’ SNPs (ie, rs73885319 and rs60910145 in G1 and rs71785313 in G2). The *Axiom Genome-Wide Pan-African Array Set* is currently the first array optimized for coverage of multiple African ancestry populations, and it maximizes coverage of common and rare variants in population of Yoruba, Luhya, and Maasi ancestry and admixed populations with West African ancestry. Its coverage of common and rare allele is ≥90% in Yoruba and ≥85% in Luhya and Maasi. The array includes over 2,000,000 SNPs selected from HapMap, 1000 Genomes, the Southern African Genomes Projects, the Sanger Center Cancer Gene Census, and the National Human Genome Research Institute Catalog of Published Genome-Wide Association Studies, and the SNPs include important disease and biological categories such as coding SNPs, pharmacogenomics genes, cardiovascular genes, major histocompatibility complex genes, and immune and inflammation pathway genes.

### Limitations: Potential Pitfalls and Alternatives

A major concern is getting funds to execute a study of this nature. However, through application for grants, it is hoped that there would be light at the end of the tunnel.

There is also a concern that there may be an insufficient sample size of children with RPS that would serve as cases. Although this may impact on statistical significance of predictor variables in regression analyses, this limitation will be surmounted by extending the study period until a satisfactory number of 50 is attained.

The pitfall of possible attrition at 2 weeks and 6-month time points will be reduced by ensuring that only parents or caregivers who fully understand the study protocol and are willing to comply with it are recruited into the study in the first place. A modest monetary incentive would also be provided when parents or caregivers bring their children at these two time points. Traceable home addresses and mobile telephone numbers of the participants would be gotten, and close contact would be maintained by periodic telephone calls.

In Nigeria, as in many sub-Saharan African countries, political and economic instability can hamper longitudinal biomedical research via incessant strikes and erratic essential services such as electricity. The engagement of the researcher in other large-scale studies would enable him to have access to the uninterrupted electricity supply already put in place by the IFAIN. Biological specimens requiring refrigeration (including −80˚C freezers) will be kept at the IFAIN laboratory service in Abuja, pending the appropriate time of shipment of such specimens.

All genetic data will be checked for consistency, and outliers will be examined. Genotypic error will be examined by including blind replicates to assess assay reproducibility and the assessment of Hardy-Weinberg (H-W) proportion for each SNP. Reproducibility of the assay using replicate samples will be assessed using the kappa statistic. SNPs showing considerable discordance (kappa ˂.9) will be discarded from subsequent analyses. Allele frequencies for each SNP will be computed and tested for departures from the H-W proportions. SNPs that persist out of H-W proportions after testing for genotyping error will be kept in the analyses as they may provide valuable insight into population ancestry, or signal a genome region for which the study sample is biased or is under selection pressure.

## Appendix

Multimedia Appendix 1 The researcher's capacity to conduct this study.

Multimedia Appendix 2 The conceptual framework of the study.

Multimedia Appendix 3 The informed consent form.

Multimedia Appendix 4 Questionnaire.

Multimedia Appendix 5 The flowchart of the study's four steps.

Multimedia Appendix 6 The power chart to show the strength of association.

## Abbreviations

ANG II: angiotensin II
APN: acute pyelonephritis
APOL1: apolipoprotein L1
CFU: colony-forming unit
CKD: chronic kidney disease
CRP: C-reactive protein
CXCL: chemokine ligand
DMSA: dimercaptosuccinic acid
EPU: emergency pediatric unit
ESR: erythrocyte sedimentation rate
ESKD: end-stage kidney disease
ESRD: end-stage renal disease
FSGS: focal segmental glomerulosclerosis
HIVAN: HIV-associated nephropathy
IFAIN: International Foundation Against Infectious Diseases in Nigeria
IFN-γ: interferon-γ
IRF3: interferon regulatory factor 3
MCUG: micturating cystourethrogram
NS: nephrotic syndrome
PCT: procalcitonin
PRR: pattern recognition receptor
RA: research assistant
RBUS: renal and bladder ultrasound
RPS: renal parenchymal scarring
SNP: single nucleotide polymorphism
TGF-β: transforming growth factor β
TLR4: toll-like receptor 4
TNF-α: tumor necrosis factor-alpha
UTI: urinary tract infection
VEGF: vascular endothelial growth factor
VUR: vesicoureteral reflux
WBC: white blood cell
