# Supplementary material for: A Putative Role of Apolipoprotein L1 Polymorphism in Renal Parenchymal Scarring Following Febrile Urinary Tract Infection in Nigerian Under-Five Children: Proposal for a Case-Control Association Study
Source: JMIR Res Protoc. 2018 Jun 14;7(6):e156. doi: 10.2196/resprot.9514 (PMC6024104; doi:10.2196/resprot.9514)
Supplement: Multimedia Appendix 1 [file resprot_v7i6e156_app1.pdf]

Conceptual framework

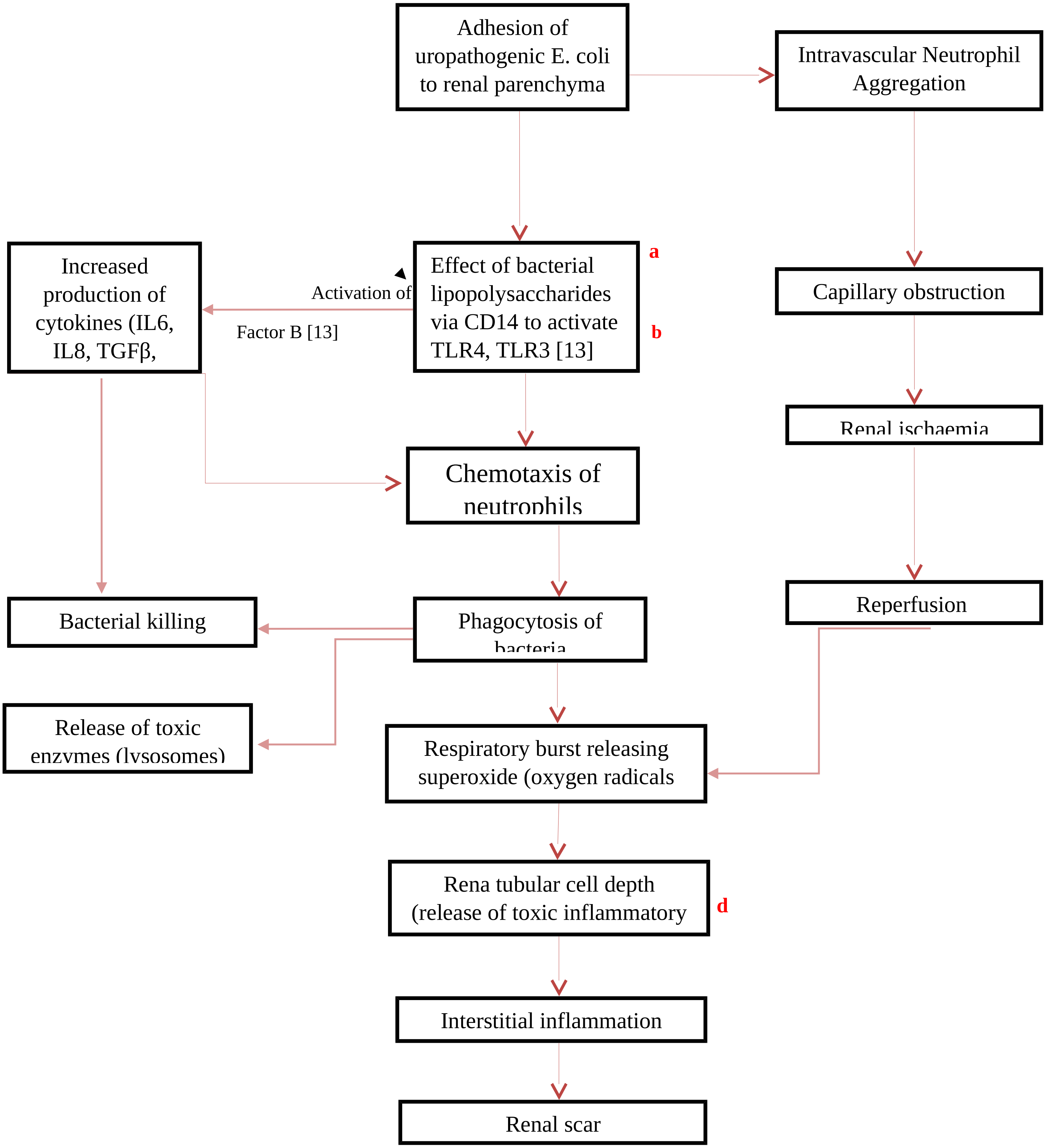

NB:

TLR4: Toll like receptor 4

TLR3: Toll like receptor 3

IF-γ: Interferon gamma

TNF-α: Tumor necrosis factor – alpha

TGFβ: Transforming growth factor - beta

VEGF: Vascular Endothelial growth factor

IFR3: Interferon regulator factor 3

IL6: Interleukin 6

IL8: Interleukin 8

Expression of APOL 1 in renal tubular cells, podocytes, vascular endothelial cells can be increased because evidence exists to support that APOL 1 expression is increased in human embryonic umbilical vein endothelial cells [56] following exposure to lipopolysaccharide.

The toll like receptors that are activated by bacterial lipopolysaccharide also increase the expression of APOL 1 when stimulated by double stranded RNA TLR3 agonist [46].

Interferon _γ and tumor necrosis factor _α are cytokines produced following activation of transcription factor nuclear factor β (μF _ Kβ). These cytokines are also known to up-regulate the expression of APOL 1 [57].Interferon regulating factor 3 (IRF3) is another cytokine produced via NF_Kβ activation, which is also involved in increased APOL 1 expression [46]

Increased expression of APOL 1 in renal tubular epithelial cells resulting in deaths of these tubular cells via APOL 1 mediated apoptosis resulting in deleterious circle of tubular atrophy, cytolytic events and renal scarring [55, 56]
