# Supplementary material for: A Putative Role of Apolipoprotein L1 Polymorphism in Renal Parenchymal Scarring Following Febrile Urinary Tract Infection in Nigerian Under-Five Children: Proposal for a Case-Control Association Study
Source: JMIR Res Protoc. 2018 Jun 14;7(6):e156. doi: 10.2196/resprot.9514 (PMC6024104; doi:10.2196/resprot.9514)
Supplement: Multimedia Appendix 2 [file resprot_v7i6e156_app2.pdf]

## Appendix II. Questionnaire

### A. Sociodemographic

1. Age: (in months) as at last birthday…………
2. Gender: Male …… Female
3. Place of residence: Urban………Rural………
4. Ethnicity (STATE IT)…………………………..
5. Socioeconomic status of the household:

   (I) Senior Public Servants, Professionals, Managers, large scale traders, businessmen and contractors
   (II) Intermediate grade public servants and senior school teachers
   (III) Junior school teachers, professional drivers, artisans
   (IV) Petty traders, laborers, messengers
   (V) Unemployed, full-time housewife, students and Subsistence farmers.

6. Phone number of parents: State it…………………………………………………………..

### B. Past medical history

7. Prior use of antibiotics in the last 2 weeks: Yes…. No……

8. Past history of UTI: Yes……. No……

9. Family history of recurrent UTI: Yes ………. No………

10. Familyhistory of congenital anomaly of the urogenital tract: Yes …… No……

11. State the congenital anomaly if present………………………

12. Past history of worm infestations: Yes………… No……….

13. Past history of constipation: Yes………No…………

14. History of breastfeeding in the first 6 postnatal months: Yes ………..No……….

### C. Presenting symptoms

15. Jaundice: Yes… No…..

16. Poor feeding: Yes ……….. No……….

17. Vomiting: Yes………. No………..

18. Diarrhoea:   Yes………No………..

19. Irritability:  Yes………No……….

20. Strong smelling urine:   Yes………  No……….

21. Abdominal pain:  Yes ………   No……..

22. Flank/back pain:  Yes ………..   No………….

23. Irritability:   Yes …………..No…………..

24. Dysuria:   Yes………..  No…………

25. Frequency of urination:    Yes……..  No……….

26. Dribbling of urine:   Yes………. No……….

27. Poor stream of urine:   Yes………. No…………

28. Straining to void:  Yes……………No…………..

29. Duration of fever in hours: State it…………………

**D.  Signs**

30. Acutely ill-looking:    Yes ………. No………

31. Temperature: State the degree in Celcius……………..

32. Weight (kg): State it……………………..

33. Height or Length (cm): State it………………………

34. Mid-arm circumference (cm): State it………………………………

35. Occipitofrontal circumference (cm): State it……………………………

36. WAZ score: State it….

37. WHZ score: State it…………..

38. HAZ score: State it………………………

39. Tenderness of the flank or costovertebral angle: Yes……… No…….

40. Suprapubic tenderness: Yes………….No…………

41. Abdominal tenderness: Yes…………….No…………

42. Circumcision (for male infant): Yes……….No…………

43. Signs of irritation on the external genitalia: Yes……..No……….

44. Pinworms: Yes……….. No……………

45. Vaginitis: Yes…………No…………

46. Trauma, or sexual abuse suspicion noted on genitals: Yes…… No………

**E. Co-morbidities**

*47.* Malaria: Yes ………….No……….

*48.* If Malaria is Yes: State the pluses………………..

*49.* Sepsis: Yes……..No……

*50.* If Sepsis is Yes: State the culture organism from the blood

*51.* If Sepsis is Yes: State the antimicrobial sensitivities…………………….

*52.* If Sepsis is Yes: State the antimicrobial resistance………………………

*53.* Upper respiratory tract infection/otitis media: Yes……No……..

*54.* Pneumonia: Yes…………No…………….

*55.* Nephrotic syndrome: Yes………No……………

*56.* Viral exanthema: Yes ……..No…………….

*57.* Malignancies: Yes ……….No………….

*58.* If Malignancies is Yes: State it…………………………

*59.* Sickle cell disease: State the genotype………………………….

*60.* HIV:  Yes     No…..

*61.* Hepatitis B: Yes….. No……

*62.* Hepatitis C: Yes…….. No……………..

### *F.* Collect the following biological specimen

63. Urine sample will be collected as per the method relevant to the age of the child: Collected…………………

64. Collect2 mLs of blood for automated complete blood count, polymorphonuclear cell counts, ESR, C-reactive protein, procalcitonin, tumour necrosis factor-α, interferon-γ, HIV and haemoglobin genotypes: Collected……………………………….

65. Collect 1.5mls of blood for DNA Extraction: Collected……………………………..
