# Supplementary material for: A Putative Role of Apolipoprotein L1 Polymorphism in Renal Parenchymal Scarring Following Febrile Urinary Tract Infection in Nigerian Under-Five Children: Proposal for a Case-Control Association Study
Source: JMIR Res Protoc. 2018 Jun 14;7(6):e156. doi: 10.2196/resprot.9514 (PMC6024104; doi:10.2196/resprot.9514)
Supplement: Multimedia Appendix 3 [file resprot_v7i6e156_app3.pdf]

Appendix III: Radioloigal result

1. **Renal and bladder ultrasound scan:**

………………………………………………………………………………………

………………………………………………………………………………………

………………………………………………………………………………………

………………………………………………………………………………………

………………………………………………………………………………………

………………………………………………………………………………………

………………………………………………………………………………………

……………………………………………………………………………

2. **Micturating cystourethrogram scan**

……

……

……

……

……

……

……

……

……

……

………

3. **Dimercaptosuccinic acid scan**

………………………………………………………………………………………

………………………………………………………………………………………

………………………………………………………………………………………

………………………………………………………………………………………

………………………………………………………………………………………

………………………………………………………………………………………

………………………………………………………………………………………

………………………………………………………………………………………

………………………………………………………………………………………

……………………………………………………………………………………
