# Supplementary material for: A Putative Role of Apolipoprotein L1 Polymorphism in Renal Parenchymal Scarring Following Febrile Urinary Tract Infection in Nigerian Under-Five Children: Proposal for a Case-Control Association Study
Source: JMIR Res Protoc. 2018 Jun 14;7(6):e156. doi: 10.2196/resprot.9514 (PMC6024104; doi:10.2196/resprot.9514)
Supplement: Multimedia Appendix 4 [file resprot_v7i6e156_app4.pdf]

Flow chart of study steps

STEP 1: Enrolment

Screen to confirm:
- Study eligibility (febrile ≥1 month - 5 years old)
- Apply inclusion and exclusion criteria
- Provide information to the parents/caregivers about the study
- Administer written informed consent

STEP 2: For enrolees while on admission at emergency paediatric unit of national hospital

a. Obtain information on:
- Ethnicity
- Age
- Gender
- Birth and pregnancy history
- Past medical history
- Family history
- Presenting symptoms
- Co-morbid history
- Complete physical examination

b. Collect biological specimens:
- Urine for urinalysis, microscopy culture and sensitivity
- .5mls of blood for complete blood count, erythrocyte sedimentation rate, C-reactive protein, procalcitonin, tumour necrosis factor-α, interferon-ϒ, HIV, sickle cell screen
- 2.5mls of blood for apolipoprotein L one DNA analysis

c. Do renal and bladder ultrasound scan (RBUS)

STEP 3: A 14-day follow up on enrolees with confirmed febrile urinary tract infections

Do micturating cystourethrogram If RBUS in step 2 shows hydronephrosis, scaring, high grade reflux or obstructive uropathy or other congenital anomalies of the kidney and urinary tract

STEP 4: A 6-month follow-up on enrolees with confirmed febrile urinary tract infections having no vesicoureteral reflux (VUR) or any other congenital anomalies of the kidney and the urinary tract

Do a dimercaptosuccinic acid (DMSA) scan on anomalies with confirmed febrile urinary tract infections having no VUR
