# Supplementary figures and images for: A Putative Role of Apolipoprotein L1 Polymorphism in Renal Parenchymal Scarring Following Febrile Urinary Tract Infection in Nigerian Under-Five Children: Proposal for a Case-Control Association Study
Source: JMIR Res Protoc. 2018 Jun 14;7(6):e156. doi: 10.2196/resprot.9514 (PMC6024104; doi:10.2196/resprot.9514)

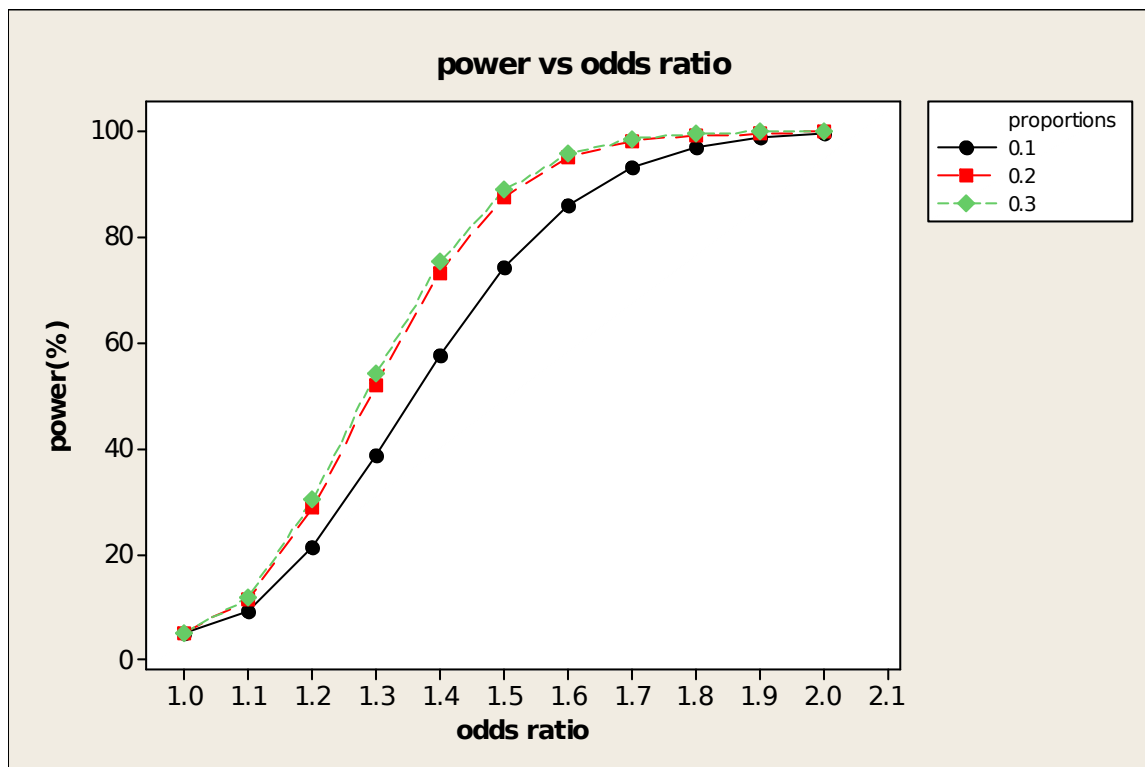

Supplement: Multimedia Appendix 5 [file resprot_v7i6e156_app5.pdf]

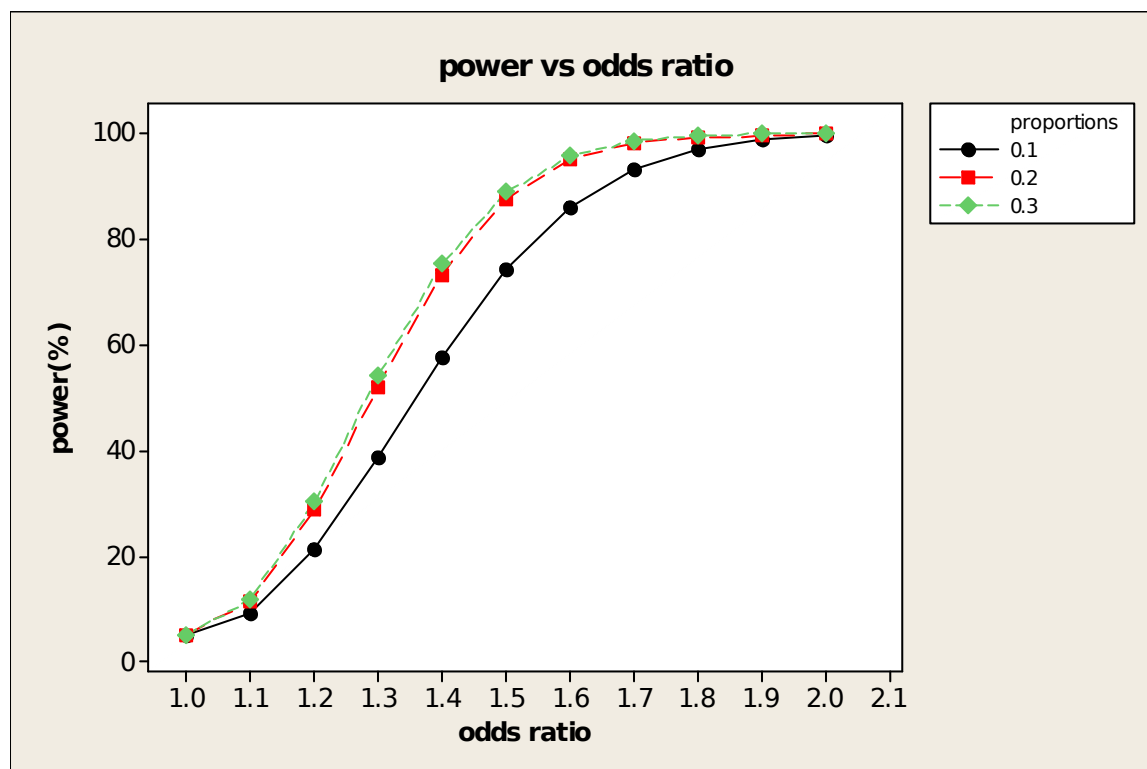

Supplement: Multimedia Appendix 6 [file resprot_v7i6e156_app6.pdf]
